# Engineering Cellular Photocomposite Materials Using Convective Assembly

**DOI:** 10.3390/ma6051803

**Published:** 2013-05-07

**Authors:** Jessica S. Jenkins, Michael C. Flickinger, Orlin D. Velev

**Affiliations:** 1 Department of Chemical and Biomolecular Engineering, North Carolina State University, Raleigh, 911 Partners Way, NC 27695, USA; E-Mails: jsjenkin@ncsu.edu (J.S.J.); odvelev@ncsu.edu (O.D.V.); 2 Golden LEAF Biomanufacturing Training and Education Center (BTEC), North Carolina State University, 850 Oval Drive, Centennial Campus, Raleigh, NC 27695, USA

**Keywords:** cellular photocomposites, continuous convective-sedimentation assembly, colloidal coatings, artificial leaves, biocomposite materials

## Abstract

Fabricating industrial-scale photoreactive composite materials containing living cells, requires a deposition strategy that unifies colloid science and cell biology. Convective assembly can rapidly deposit suspended particles, including whole cells and waterborne latex polymer particles into thin (<10 µm thick), organized films with engineered adhesion, composition, thickness, and particle packing. These highly ordered composites can stabilize the diverse functions of photosynthetic cells for use as biophotoabsorbers, as artificial leaves for hydrogen or oxygen evolution, carbon dioxide assimilation, and add self-cleaning capabilities for releasing or digesting surface contaminants. This paper reviews the non-biological convective assembly literature, with an emphasis on how the method can be modified to deposit living cells starting from a batch process to its current state as a continuous process capable of fabricating larger multi-layer biocomposite coatings from diverse particle suspensions. Further development of this method will help solve the challenges of engineering multi-layered cellular photocomposite materials with high reactivity, stability, and robustness by clarifying how process, substrate, and particle parameters affect coating microstructure. We also describe how these methods can be used to selectively immobilize photosynthetic cells to create biomimetic leaves and compare these biocomposite coatings to other cellular encapsulation systems.

## 1. Introduction

Fabricating industrial-scale composite materials as ordered arrays of adhesive polymer particles and photosynthetic living cells that sustain viability during film formation (drying) and are mechanically stable when rehydrated, requires a deposition strategy that unifies colloid science and cell biology. Convective assembly is one method that can be modified to rapidly deposit a variety of particles, including living cells and adhesive latex particles into <10 µm thick organized films with engineered adhesion, composition, thickness, and particle packing. These cellular photocomposite coatings have a wide variety of future applications, including photobiological hydrogen production, oxygen evolution or utilizing the biosynthetic capabilities of non-growing cells to assimilate carbon dioxide and generate liquid fuel precursors [1,2,3]. Cellular photocomposites could be fabricated to contain genetically engineered cells to efficiently generate clean fuels from sunlight, water, carbon dioxide and industrial byproducts [4,5,6], as non-toxic alternatives to antifouling paints [7,8,9], or to eliminate fouling and contamination through cell shedding, digestion, or antibiotic secretion [10]. 

Hyperthermophiles [11], prokaryotes, and yeast [12,13] can be preserved and stabilized when deposited in <50 µm thick adhesive films of randomly ordered cells and partially coalesced low T_g_ waterborne acrylate, polystyrene or co-polymer particles. The entrapped cells retain viability during film drying, storage [14], and rehydration and are reactive without outgrowth. Resting cells [15], cells encapsulated in agar [16], alginate [17], and silica [18] or codeposited with adhesive waterborne latex emulsions have been shown to be more stable and often more reactive than planktonic cells [19]. However, only colloid-based latex biocomposite adhesive coatings can stabilize desiccated cells without loss of reactivity, concentrate (500 to 1000-fold) cells to a very high volume fraction, and when containing appropriate carbohydrates maintain the integrity of microbial membranes during drying [14,20]. Once rehydrated, these cellular composites have been shown to stabilize cellular photoreactivity and protein synthesis without outgrowth for thousands of hours [2]. Photocomposite light absorption and light scattering properties could be significantly improved in the future by depositing a variety of different cells with complementary light absorption and carbon assimilation pathways optimized by synthetic and systems biology methods. Therefore developing deposition methods to control cell and particle packing and coating thinness will provide powerful techniques for assembling multi-layer photocomposites with new functionality analogous to, but potentially more advanced than, mature plant cells in natural leaves [16]. 

**Figure 1:**
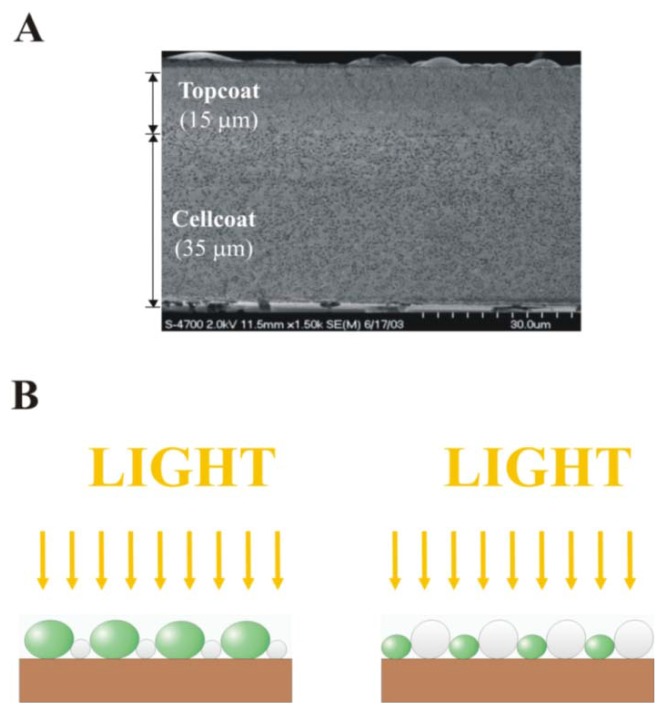
Comparison of (**A**) a randomly oriented biocomposite latex coating [24]; and (**B**) thin ordered cellular photocomposite monolayer coatings. Ordered monolayer coatings of polymer particles and photoreactive cells (shown in white and green, respectively) enable uniform light distribution (yellow arrows) and nutrient delivery to all cells.

Cellular reactivity is also a function of the support matrix’s thinness and nano porosity which are formed during film formation and drying [21]. Latex coatings, unlike hydrogel matrices, maintain adhesiveness and structural integrity when wet or dehydrated and can be readily engineered for improved nanoporosity by altering polymer particle chemistry or co-depositing carbohydrates and glycerol. These porogen and osmotic stabilizing additives protect the entrapped cells from osmotic stress by arresting polymer particle coalescence during coating formation. However, although a variety of coating methods and waterborne latex formulations have been shown to preserve microbial viability and reactivity during film formation and dehydration, new deposition methods are needed to make ordered monolayers (one particle or one cell thick) to engineer multi-layer coating microstructure for optimal composite reactivity [22,23]. Monolayers have improved nutrient diffusion, uniform cellular illumination and reduced cell to cell shading and light scattering properties, thus overcoming the mass transfer and optical limitations of thicker hydrogels, drawn down latex coatings, and bioceramics (Figure 1). The function of multi-layered composites cannot be engineered without first understanding the properties of monolayer (one cell thick) cellular composites.

Here we review the non-biological convective assembly literature, with an emphasis on how this crystalline colloid array deposition method has evolved from a batch process limited to the creation of small surface area monodispersed particle arrays to its current state as a continuous process (continuous connective-sedimentation assembly or CCSA) capable of fabricating larger composites of not only polymer particle arrays but also biocomposite coatings from a diverse range of particle suspensions. CCSA can be scaled-up to make larger surface area coatings by simply changing the size of the coating knife and substrate, giving the method greater industrial promise than other common, thin-film colloidal assembly techniques like spin coating and dip coating that require both excess coating suspension and simultaneous scaling of multiple parameters (see [25] for a detailed review of the latter assembly techniques). Further development of the convective assembly method will help solve the fundamental challenges of engineering multi-layered cellular composite coatings with high reactivity, stability, and robustness by clarifying how multiple process, substrate, and particle parameters affect coating microstructure. We also describe how this method can be modified to precisely fabricate arrays of live cells on various substrates to create cellular photocomposite materials for future use in biomimetic artificial leaves and compare the requirements of biocomposite coatings to other cellular encapsulation systems used in biocatalyis. 

## 2. Composite Cellular Coating Fabrication Methods 

Methods to generate <75 µm thick adhesive latex plus microbial biocoatings were pioneered by Flickinger and collaborators using simple drawdown Mayer rod coating methods, however the lower limit of coating thinness of this method is ~10 µm [25]. This group demonstrated that low T_g_ acrylate latex binder emulsions can form nanoporous coatings that retain adhesion to coating substrates when rehydrated because the latex particles partially coalesce and “glue” the cells to the substrate. During film drying, carbohydrate additives partially arrest particle coalescence forming nanopores surrounding the micron-size cells [2,3,11]. This method is capable of fabricating biocomposite coatings containing a very high density of a wide variety of microorganisms without loss of coating mechanical stability (delamination, cracking) or impairment of microbial viability, protein synthesis, and reactivity following rehydration [20,21]. 

This collaborative effort at the University of Minnesota used a Meyer rod draw down coating method to deposit composites of randomly ordered microbial cells and 150 nm to 850 nm diameter latex particles in 5 to 100 cm^2^-scale patches, strips, or sheets on flexible polyester substrates with a range of thicknesses from 10 to 250 µm in either monolayer or bilayer biocatalytic coatings [2,3,11,21,26]. Microbe viability is preserved during film formation and drying by the addition of osmoprotective carbohydrates (that also arrest polymer latex coalescence into a non-porous film) by forming carbohydrate glasses surrounding the cells during drying [14,20]. Control of drying conditions is critical to retain cellular reactivity. This method relies on low T_g_ acrylate co-polymer or non-film forming polymer particle blends plus carbohydrates to alter compaction and arrest coalescence to generate nanoporosity [27]. However, this coating strategy is limited by wire diameter to coatings of >10 µm thick. 

**Figure 2:**
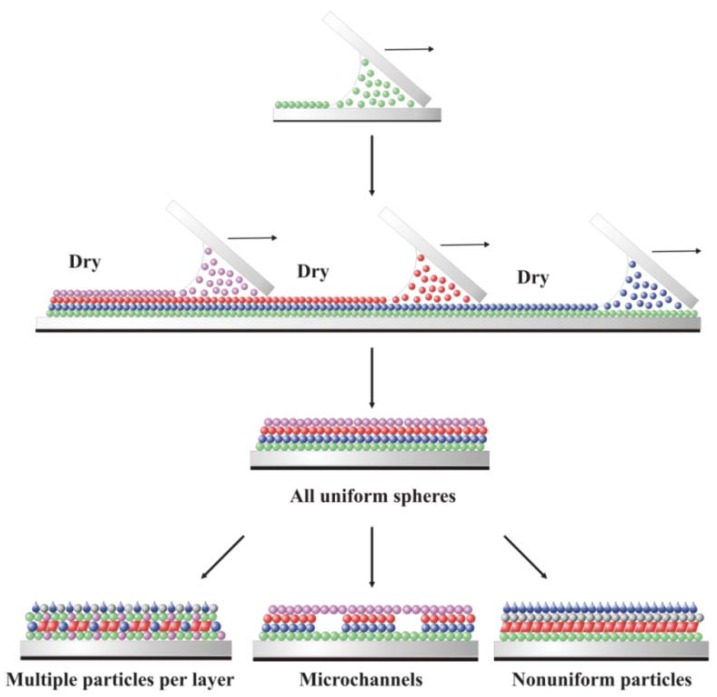
Schematic of four composite coatings deposited using sequential layer-by-layer convective assembly to form a composite device. Each color represents a different type of particle or cell. Each new layer is deposited after the underlying layer has dried.

Fabrication of reactive multi-layer coatings for prototype devices utilizing adhesive coatings of a variety of genetically optimized prokaryotic, eukaryotic and plant cells [2,3,11,21,22,24] will require new methods of polymer particle plus live cell deposition to investigate the reactivity of a single cell layer. Once the properties of composite monolayers are understood, layer-by-layer (LBL) coating methods will be needed to fabricate multi-layered and channeled systems with tailored diffusion, light trapping, gas harvesting and reactivity [2] (Figure 2). As such, understanding how to reliably fabricate monolayer arrays of polymer particles and live cells on rigid and flexible substrates is of fundamental and practical importance [27].

In order to extend these early cellular composite coating concepts, to investigate the fundamentals of cell-cell and cell-particle interactions and to engineer coating microstructure, convective assembly deposition was investigated in a collaboration between Flickinger and Velev [23,27] as a method of coating cellular monolayers or very thin composite materials. Convective assembly combines fluid evaporation, particle transport via fluid flow, and associated meniscus motion to rapidly and controllably deposit a diverse range of microspheres and cells into thin (<10 µm) highly uniform films [28,29,30,31,32,33,34]. Our preliminary results summarized here suggest that further refinement of convective assembly could lead to rapid, scalable, and repeatable fabrication of well-ordered, multi-layer bio-mimetic arrays of living cells and polymer particles on an industrial scale.

**Figure 3:**
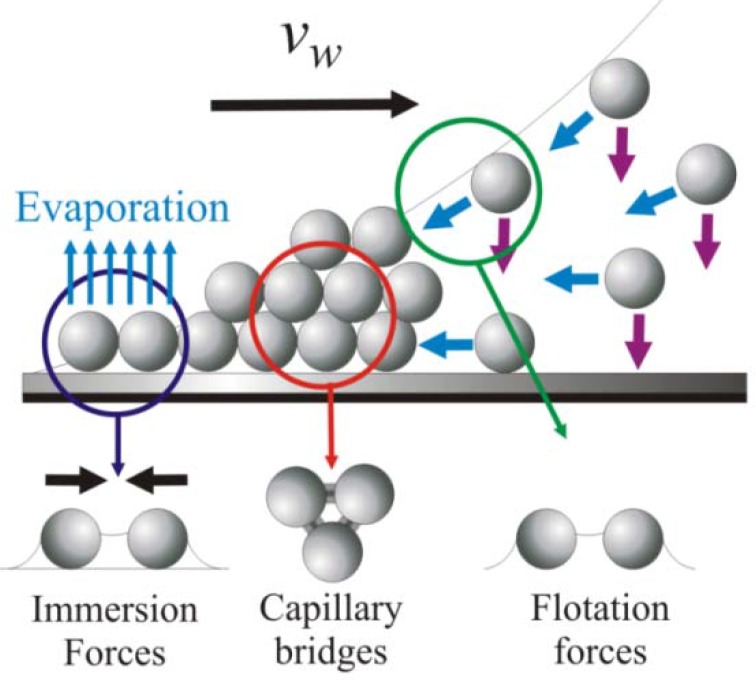
The convective assembly process. Blue and purple arrows denote particle convection and sedimentation in meniscus, respectively [35].

In convective assembly, the particle or cell assembly process begins at the periphery of an evaporating fluid film when the film’s height becomes thinner than the diameter of the suspended particles [22]. The menisci formed around these particles induce strong, long-range capillary forces that pull neighboring particles together into two-dimensional nuclei (Figure 3). 

The particle clusters travel with the liquid flux from the bulk suspension to the substrate-air-liquid contact line at the drying front as the fluid evaporates, resulting in the formation of closely packed arrays and subsequent propagation of the coating area (Figure 3) [36,37]. The coating growth rate, *v_c_*, is related to the fluid evaporation rate and particle volume fraction by the equation

(1) $$v_{c}=\frac{\beta \;j_{e}\;l\;\varphi }{h\;\left(1-\varepsilon \right)\;\left(1-\varphi \right)}$$

where *β* is a particle-particle interaction parameter; *j_e_* is the evaporation rate; *l* is the drying length; *φ* is the volume fraction of the particles in suspension; *h* is the height of the deposited coating; and *ε* is coating porosity [35,38]. Values of *β* vary between 0 and 1 and depend on particle-particle and particle-substrate interactions; for low volume fraction and electrostatically stable particles, *β* *≈* 1. Once *v_c_* is determined, the length of the thin film where deposition occurs by convection can be calculated using a material flux balance:

(2) $$L_{film}=\frac{v_{w}d_{cell}\left(1-\varepsilon \right)\left(1-c_{i}\right)}{\beta j_{e}c_{i}}$$

where *v_w_* is the deposition rate and equal to *v_c_* at steady state; and *c_i_* is the concentration of the bulk suspension at that particular time [35]. 

**Figure 4:**
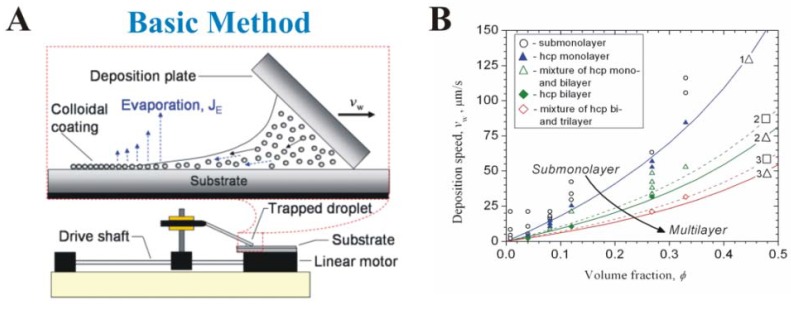

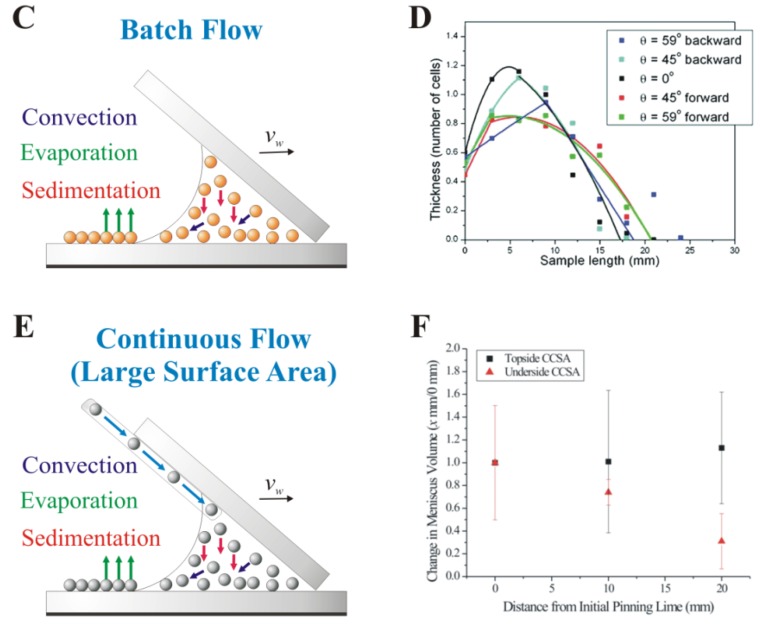
Evolution of the convectively-assembled coating technique. Mechanistic parameter effects for (**A**,**B**) evaporative convective assembly [30]; (**C**,**D**) convective-sedimentation assembly [22]; and (**E**,**F**) continuous convective-sedimentation assembly [39].

Velev’s group adapted convective assembly from a slow, randomized process occurring at the edge of a stationary droplet [36] to a rapid, controllable process occurring at the boundary of a dynamic fluid. Prevo and Velev reported a convective assembly technique for rapid and controllable deposition of coatings from polymer microspheres (1.1 µm) or gold nanospheres (~12 nm) (Figure 4A) [30]. Up to 30 µL of suspension containing polymer particles at high volume fraction (0.9%–35% w/v) or gold nanospheres (0.01%–0.3% w/v) is trapped between a horizontal substrate and an inclined top plate or coating knife. Pushing the coating knife top plate at a constant rate along the long axis of the bottom plate by a linear motor spreads the suspension from the meniscus into a thin film across the horizontal substrate, leading to coating fabrication on the substrate by evaporative convective assembly [22]. Both the coating thickness and structure of the deposited particle layers are readily adjusted by altering the suspension volume fraction, coating knife speed, or knife (blade) angle together with coating knife speed, allowing for precise control of particle packing and coating thickness (Figure 4B) [29,30].

Jerrim and Velev realized that evaporative convective assembly alone cannot directly generate high-quality cellular coatings due to cell sedimentation onto the substrate below the moving meniscus during coating deposition (Figure 4C). Yeast are at least five times larger than the other types of particles previously deposited using evaporative convective assembly [30,40,41,42] leading to cell sedimentation that significantly alters coating uniformity [22]. To combat this process, they created convective-sedimentation assembly, a modified version of the deposition technique that controls sedimentation (and coating thickness) by using variations in the tilt (relative to a horizontal surface) of the coating apparatus to direct settling cells toward (*forward* in Figure 4D) or away from (*backward* in Figure 4D) the drying front during fabrication [22]. Using this adaptation they obtained thin (<2 cell layers), close-packed yeast coatings in 15–45 minutes. 

However, batch convective-sedimentation assembly lacks utility on an industrial scale because each particle array’s total surface area is limited by the finite amount of suspended particles delivered to the drying front by the continuously depleted coating knife meniscus volume. We recently overcame this shortcoming by developing a process of continuous convective-sedimentation assembly (CCSA) which uses inline injection to create larger surface area and longer thin films by constantly dispensing suspended, uniformly charged particles (or charged particle and live cell suspensions) to the meniscus (Figure 4E) [23]. Coating microstructure can be controlled by varying the suspension delivery mode between topside CCSA, in which suspension flows through a capillary from a fluid reservoir to the front of the meniscus along the coating knife’s topside, and underside CCSA, in which suspension flows from a fluid reservoir through tubing fixed to the back of the meniscus along the knife’s underside. Each mode achieves a certain meniscus volume and characteristic particle delivery to the drying front, enabling microstructure control by varying the total number of particles available for deposition (Figure 4F) [23]. Because CCSA enables continuous coating fabrication without loss of deposition speed, this technique, with further refinements to optimize particle packing, is promising for generating monolayers or very thin (1 to 3 cell thick) live cell plus latex polymer composite coatings for applications where a large, highly biocatalytic or photoreactive surface area is required. 

## 3. Early Cellular and Particle Array Composite Coating Systems

Microbial latex coatings were introduced in the 1980s by Lawton, Bunning, and Flanagan, who coated solid particles, nylon mesh, membranes, and silica particles with polydispersed acrylate/vinyl acrylate copolymers [43,44,45,46,47]. Cantwell first reported the use of polymer blends of hard and soft polymer particles for microbial entrapment, but did not fabricate thin coatings—only flocculates, 1–2 mm aggregates, and 2 mm diameter fibrils [21,48]. Martens and Hall reported methylmethacrylate and butyl acrylate polymer coatings of photosynthetic *Synechococcus* on a carbon electrode, creating a functional biomimetic device that showed cell viability of “nearly 100%” with photoreactivity after rehydration and exposure to light [21,49]. These early cellular microbial coating systems suffer from low coating permeability, weak mechanical stability (delamination from the support particles), poor control of coating thickness, uniformity, loss of reactivity and lack of defined coating microstructure (porosity, pore structure) characterization among other limitations [21]. 

**Figure 5:**
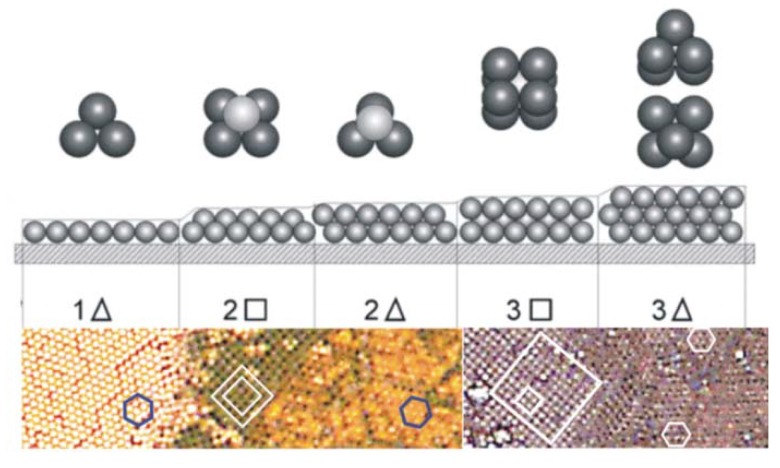
Layering transition schematic for monodisperse microspheres deposited using evaporative convective assembly. The different colors of each layer in the experimental micrographs are due to optical interference of the transmitted light; polygons are drawn to clarify the packing structures. Micrographs illustrate structures from 1.1 µm polystyrene microspheres [30]. Transition schematic originally reported by [50,51].

Overcoming these restraints to reproducibly fabricate uniformly thick, consistently porous, and highly stable multi-layer coating systems requires both structural and reactivity optimization or each layer and the composite structure. Concurrent optimization of both parameters involves systematic investigation of numerous variables. We therefore simplified the optimization by initially focusing on deposition of monodispersed latex particle monolayers. This eliminated coating reactivity (by removing all cell properties such as size, charge, age, *etc*.). Prevo and Velev were the first researchers to identify the critical parameters responsible for rapid and controlled deposition of crystalline colloidal arrays, thus enabling control over coating thickness and structure and solving the limitations of the earlier microbial latex coating systems (Figure 5) [30]. 

Using operational “phase” diagrams that correlate coating thickness and packing structure against suspension volume fraction and coating deposition speed, Prevo and Velev fabricated high-quality, convectively-assembled coatings from a diverse range of colloids, including monodispersed latex microspheres [30], untreated metallic gold nanoparticles [30,41], and dielectric nanoparticles like silica [40]. Tessier *et al.*, used convective assembly to deposit gold nanoparticles and latex spheres in a single step [48]. The latex spheres assemble into an ordered crystal array while the nanoparticles simultaneously collect in the void space of the drying array, forming a composite structure that can serve as a template for assembling gold nanoparticles into thin, porous films [52]. 

**Figure 6:**
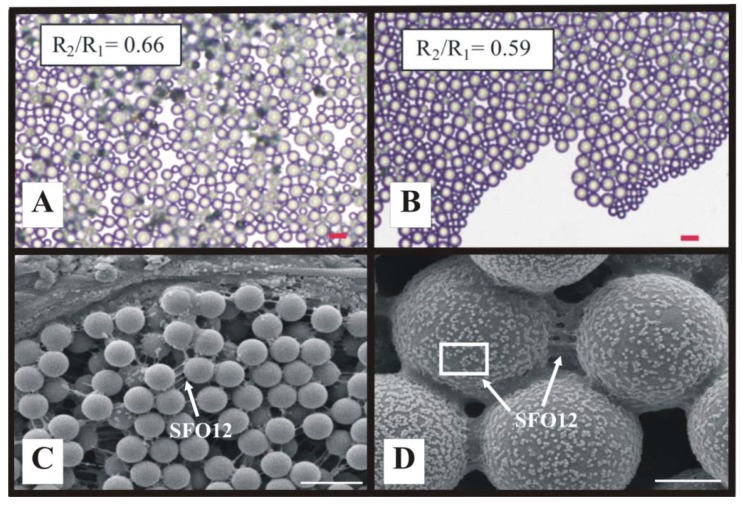
Examples of composite coatings assembled from bimodal blends of (**A**,**B**) latex polystyrene microspheres [27]; and (**C**,**D**) adhesive Rhoplex^TM^ SFO12 low T_g_ acrylate 300 nm microsphere emulsion and latex polystyrene microspheres [39]. R_2_/R_1_ is polymer particle diameter ratio. Microsphere sizes are: (**A**) 5.7 and 8.7 µm; (**B**) 5.7 and 9.6 µm; (**C**) 1.0 µm; and (**D**) 5.0 µm. The SFO12 latex emulsion microspheres aggregate on the larger polystyrene microspheres and form amorphous bridges between neighboring microspheres. Scales bars are (**A**,**B**) 20 µm; and (**C**,**D**) 2 µm.

We recently extended the previous monodispersed coating investigations to demonstrate that close-packed, convectively-assembled composite coatings can be created from bimodal latex polystyrene suspensions (Figure 6A,B) and blends of Rhoplex^TM^ SFO12 (a commercial 300 nm diameter film-forming, acrylic copolymer emulsion) and latex polystyrene microspheres (Figure 6C and Figure 6D) [27]. Each system’s particle size ratio controls the convective mixing or demixing of the suspension components during coating fabrication and thus their relative locations in the deposited coating [27].

**Figure 7:**
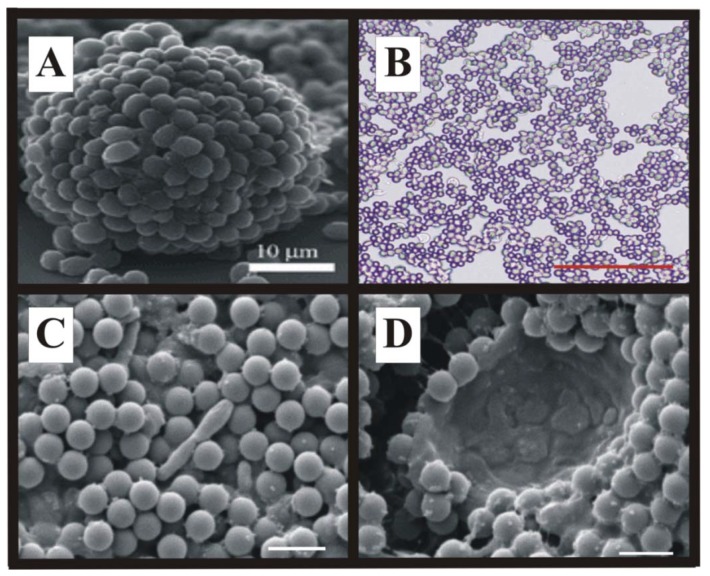
Examples of advanced biocomposite structures: (**A**) Bubble-templated “cellosomes” [55,56]; (**B**) Composite yeast-polystyrene coating [39]; (**C**,**D**) Composite coatings of photosynthetic cells, Rhoplex^TM^ SFO12, and 1.0 µm polystyrene microspheres for hydrogen evolution [39]. *Rhodopseudomonas palustris* and an artifactually collapsed *Chlamydomonas reinhardtii* (algae) are shown in C and D, respectively. Scale bars are (**B**) 70 µm; and (**C**,**D**) 2 µm.

Current fabrication efforts focus on directed, controlled, and scalable colloidal self-assembly of cells and latex microspheres into biocomposite materials with novel structures and functionalities (Figure 7) [53,54]. 

Jerrim and Velev created thin mixed layers of *Saccharomyces cerevisiae* (~5.0 µm diameter) and large (10.0 µm diameter) polystyrene microspheres by investigating the fundamental effect of cell sedimentation on the deposition process and developing a means to control the trajectories of the settling cells [22]. By rotating their coating device around a horizontal axis, they changed the position of the point at which sedimentation begins to contribute to coating thickness, creating a transition to thicker coatings—inclining the apparatus forward increased the thickness at an earlier point in the coating, improving coating uniformity while inclining the apparatus backward moves the transition farther into the coating, reducing coating uniformity [22]. We further investigated colloidal assembly of polystyrene microspheres (1.0, 5.0, and 8.7 µm) blended with yeast or photosynthetic bacteria or algae on nonporous substrates such as glass or polyester sheet. The former investigation demonstrated that simultaneous deposition of cells and latex is possible because the cells behave like simple surface-charged colloidal particles akin to the latex microspheres and that the assembly process is dominated by net particle charge [27]. 

Extending the previous work to a porous non-woven substrate coated using continuous convective-sedimentation assembly, we recently showed how reactive biocomposite coatings of either *Rhodopseudomonas palustris* (a photosynthetic purple non-sulfur bacteria) or *Chlamydomonas reinhardtii* (a unicellular green micro algae) can be fabricated from suspensions containing both non-film forming (photosynthetic cells and 1.0, 5.0, and 8.7 µm latex polymer microspheres) and film-forming laticies (Rhoplex^TM^ SFO12 emulsion) of variable sizes and charges [39]. Placing these coatings in a gas phase in contact with a small-volume liquid phase which wet the paper pores (by capillary action) and maintained cells hydrated by the liquid filled paper pores prolonged its useful life, inhibited cell outgrowth into the liquid phase and facilitated the absorption and escape of gases (such as H_2_) from the coating [1]. Variations in microsphere size and suspension composition altered coating microstructure but did not affect coating reactivity. However, despite different microstructures, all coatings retained adhesion and cellular photoreactivity after rehydration [39]. These coatings also had a higher surface-to-volume ratio than comparable alginate films [57] resulting in improved mass transfer and light distribution to the immobilized cells [58]. This partial wetting, combined with the non-woven cellulose fiber substrate’s low water uptake (2.27 × 10^−4^ ± 3.72 × 10^−5^ g/cm^2^) when completely saturated, suggests that paper or non-woven fiber supports may be robust substrates for fabricating gas-phase, photoreactive coatings from water-based cell plus latex polymer microsphere blends. The coating components adhere to the cellulose fibers without plugging the paper pores [1,39], allowing for nutrient transport from the bulk liquid phase to the immobilized cells on the surface of the paper. The open pore structure may also facilitate the absorption and evolution of gases by the coating [1,39]. 

## 4. Future Cellular Composite Materials: Self Cleaning Composites, Artificial Leaves and Biomimetic Energetic Materials 

Convective assembly allows for the selective immobilization of non-growing cells for a diverse range of applications, including foul-release or self-cleaning coatings. Fouling refers to the unwanted accumulation or formation of biomass, sludge, particulates, inhibitors or fats/oils that appreciably degrades a coating’s performance and useful life [20]. The half-life of most all reactive coatings is limited by fouling or contamination. Prior studies on fabricating self-cleaning surfaces focused on inorganic antifouling paints containing one or more of (1) heavy metals, including cadmium, chromium, silver, and zinc [9]; (2) biocides like chlorine [59,60,61] and mercury [59]; or (3) antibiotics [60,62]. Because biocides or antimicrobials may endanger human health or cause environmental damage when leached from a coating [8] and pervasive use of antibiotics promotes microbial resistance [63], these paints are restricted in many countries [9,63], suggesting the need for biological approaches to generate non-toxic, “natural” antifouling paints [64]. Fabrication of cellular composite surfaces with controlled wetting and composition that secrete antimicrobial compounds have the potential to be developed into “natural” foul-release coatings [65]. 

**Figure 8:**
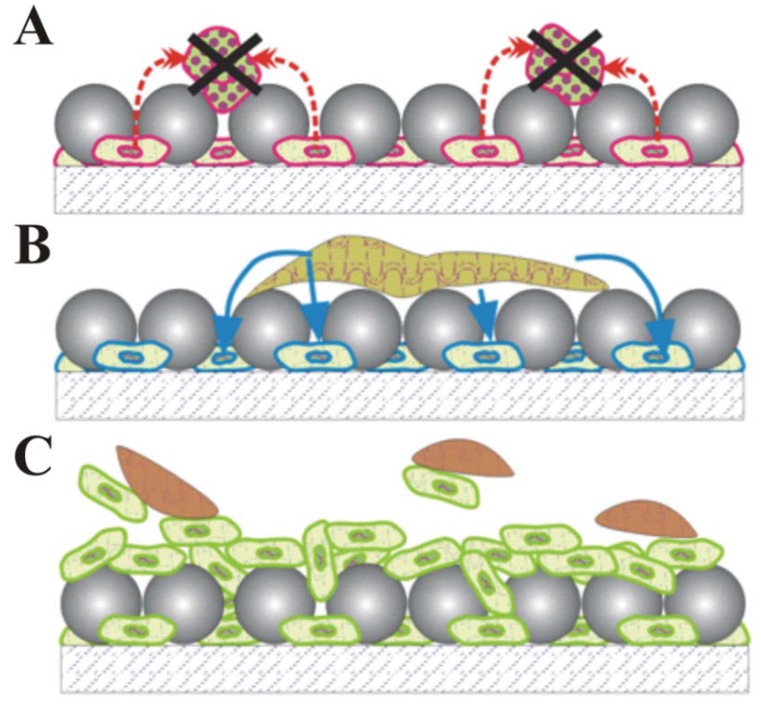
Proposed self-cleaning strategies for cellular biocomposite coatings containing polymer particles and living cells: (**A**) Bactericidal antibiotic secretion; (**B**) Contaminant digestion; (**C**) Top layer shedding and self-regeneration.

All surfaces can self-clean whenever the adhesion forces between surface debris and the surface are weaker than those between the contaminants and tangential fluid, leading to the debris being carried away in the fluid as it runs off the surface [65,66]. As such, a diverse range of inorganic and biomimetic antifouling surfaces can be fabricated by tuning the surface properties to weaken this attraction force. In contrast, cellular biocomposite coatings may eliminate surface debris through three additional mechanisms: (1) *shedding and self-regeneration*, which removes contaminants through cell shedding underneath the debris layer while simultaneously regenerating the depleted top layer by cell proliferation (Figure 8A) [22]; (2) *digestion*, which uses contaminant-targeting cells to digest contaminating particulates as they diffuse downward through the coating (Figure 8B); and (3) *secretion*, which uses bactericidal secretion of antibiotics by the cell in the composite to inhibit growth of bacteria, algae and fungi contaminants on the coating surface (Figure 8C) [10]. 

**Figure 9:**
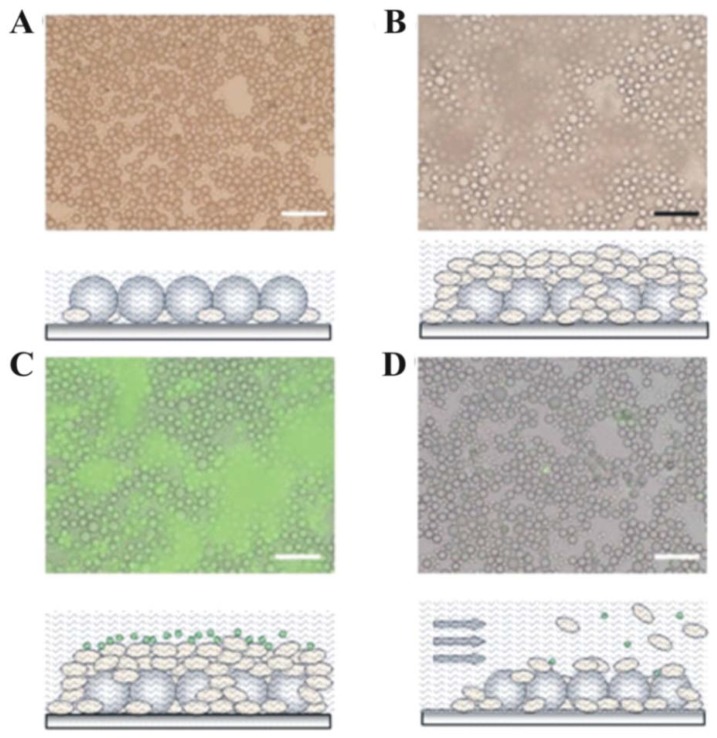
Micrographs and schematics demonstrating biocomposite (*S. cerevisiae* and latex microsphere) coatings with rudimentary self-cleaning properties. (**A**) Initial coating; (**B**) Coating after 24-hour incubation in growth medium, allowing for significant cell proliferation; (**C**) Coating with fluorescent latex artificial debris; (**D**) Coating following contaminant and cell removal with a stream of growth medium. All scale bars are 50 µm [67].

Previous preliminary results by our group suggest both the cellular composite *secretion* and *shedding and the self-regeneration* mechanisms can effectively cleanse debris-laden coatings. We demonstrated rudimentary self-cleaning by codepositing (using convective-sedimentation assembly) mixed monolayers of ~5 µm *Saccharomyces cerevisiae* yeast cells and 10 µm latex particles (Figure 9). The larger latex particles settled over the yeast cells, creating a porous topcoat that protects the cells from external fluid perturbations without hindering their access to nutrients and ability to proliferate through the latex topcoat (Figure 9B) [22]. When rinsed with a stream of growth media, the outermost cell layer sloughed off the coating surface, taking contaminating debris (simulated by fluorescent microspheres) with it (Figure 9C and Figure 9D) [22].

One cellular biocomposite coating was stained with FUN1 dye and subsequently analyzed for metabolic activity to assess the viability of the deposited cells; the presence of non-uniform fluorescence within the cells indicated metabolic activity, thus confirming the coating’s ability to self-regenerate. Burgess and coworkers demonstrated the efficacy of the secretion approach by fabricating “natural”, water-based paints that exhibited good activity against marine bacteria, barnacle larvae, and algal spores [64]. The paint formulations contained extracts from antibiotic-producing marine bacteria and a water-based resin [64]. Although field trials showed the paints had little effect on the onset and degree of macrofouling *in vivo*, likely due to rapid leaching from the coating surface, the antifouling efficacy of the laboratory assays suggests that broad spectrum antifouling can be achieved through judicious combinations of metabolites with different leaching rates from painted surfaces and antifouling activities [64]. 

Another promising use of the convective assembly technique is the fabrication of cellular light-harvesting, multi-layered structures with channels capable of mimicking the layering and vascular system of mature (non-dividing) plant cells in natural leaves—biomimetic leaves. Like plant leaves, these layered devices of non-growing cells can capture solar energy, split water into hydrogen and oxygen, and reduce atmospheric carbon dioxide into carbohydrates and other metabolites, thus synthesizing various forms of fuels from solar energy [68]. 

**Figure 10:**
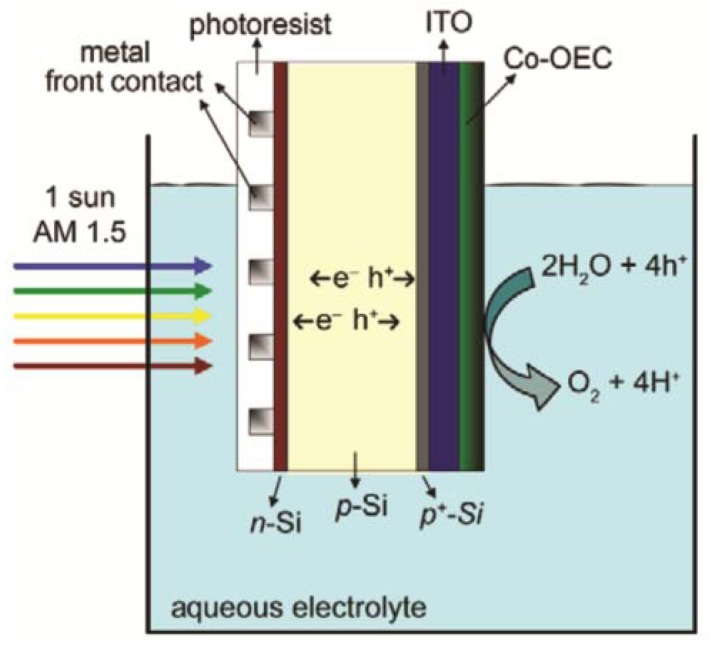
Example of an inorganic artificial leaf: A CO-oxygen evolving complex functionalized *npp^+^*-silcon single-junction photoelectrochemical cell [70].

In contrast to the cellular approach, a diverse range of non-cellular artificial leaves have been reported in the literature. Inorganic synthetic leaves have been constructed as photoelectrochemical devices that generate hydrogen by combining free electrons (generated through capture of solar energy) and protons (created through water oxidation at an anode) at a cathode (Figure 10) [69]. Some devices achieve light harvesting by mimicking the hierarchical structure of natural leaves while others imitate the natural visible-light response through dye sensitization [68]. Unfortunately all of these prototype devices have expensive components and have limited stability.

Although most non-cellular artificial leaves possess higher solar energy conversion efficiencies than many crop plants (<1% typically) and bioreactor-grown microalgae (3%) [69,71], several technical and scientific challenges to developing robust, inexpensive inorganic, or non-cellular photoreactive leaves remain. These include (1) efficient use of the entire solar light spectrum; (2) fabrication techniques that are amenable for mass production; (3) use of inexpensive, abundant materials that are readily scaled-up for bulk production; and (4) development of robust devices with lifespans of thousands of hours to years [68,69]. The non-cellular artificial leaf literature also describes attempts to immobilize or entrap photosynthetic components within support matrices, mimicking the light-harvesting pigments embedded within the thylakoid membranes of chloroplasts [72] to create biological (but non-cellular) artificial leaves. The support materials serve multiple functions, including assisting in the organization and protection of the photoreactive components, allowing for easier handling of the photoreactive device, and facilitating recycling from the reaction mixture [72,73,74]. This class of biomimetic leaves includes water-insoluble porphyrins embedded within a lignocellulosic matrix [72], platinized photosystem I protein monolayers immobilized on gold [75], and hybrid complexes of photosystem I and [Ni-Fe]-hydrogenase assembled on gold electrodes [76]. These composites, although partially biological, lack the stability of intact cells.

Genetically optimizing photosynthetic efficiency using synthetic biology in intact photosynthetic cells is an attractive less expensive alternative to immobilization of unstable photoreactive molecules and labile protein complexes because whole cell immobilization eliminates the need for expensive reagents and the cost of protein purification, simplifying the fabrication, enhancing the stability of the photosynthetic apparatus (in its native, most stable form) and thus reducing cost. Various types of cellular (whole cell) bio-mimetic leaves are reported, including those made from living cells and photoactive materials cross-linked in sol-gel synthetic polymers, sol-gel oxide ceramics, and silica gel glasses (see Table 1) [17,77,78,79,80,81,82,83]. 

**Table 1:** Examples of bio-mimetic leaves containing living cells.

Matrix	Cell system	Application	Reference
Silica	*Candida tropicalis Pseudomonas*	Phenol and PCB degradation	[77]
Silica	*Mixed bacteria*	Sulfate reduction	[78]
Sol-gel	*Escherichia coli*	Galactose production Alkane and dicylcopropyl detection	[80,81]
Ceramics	*Rhodococcos rhodochrous Aspergillus versicolor*	Phenol and glycol degradation	[83]

For instance, Su *et al.*, created living, biocomposite matrices of silica and photoreactive algae (*Chlorella vulgaris* or *Botrycoccus braunii*) or *Arabidopsis thaliana* (plant cells); the algae and plant systems exhibited photosynthetic reactivity for 70 and 30 days, respectively [84,85]. However, although mechanically robust and sufficiently porous for nutrient diffusion and cell proliferation, most cellular sol-gel ceramics cannot entrap more than 5% to 20% (w/w) of cell mass—higher concentrations of cells reduce coating stability when in contact with water [21]. Also, many silica networks shrink during lyogel drying, leading to reduced cell viability [21]. 

Cell-laden alginate gels [57,86] and hydrogels [87,88,89,90,91,92,93,94] are other possible forms of cellular composite biomimetic leaf materials. Researchers at The National Renewable Energy Laboratory have fabricated photoreactive Ca^2+^-alginate films containing *Chlamydomonas reinhardtii* (a microalgae) that evolve hydrogen for over 150 hours [57], but these coatings lack the thinness, adhesion and organization of convectively-assembled coatings. The increased thickness of hydrogels reduces productivity by hindering mass transfer and light distribution to all cells. Also, alginate films must remain wet during gel cross-linking to prevent cracking, skinning, shrinkage and other mechanical instabilities that occur when hydrogels are dried that lead to cell death. They also lack engineered adhesion [21]. In addition, hydrogels are macroporous (pores larger than microorganisms) with thin pore walls, leading to substantial cell release and outgrowth when the gels are exposed to nutrients to regenerate activity and sustain cell viability [21]. These features, combined with an inability to be stored dry or frozen without loss of cell viability [87,88], suggest hydrogel composites are not a suitable material for fabricating cellular photoabsorbers on an industrially-relevant scale. 

**Figure 11:**
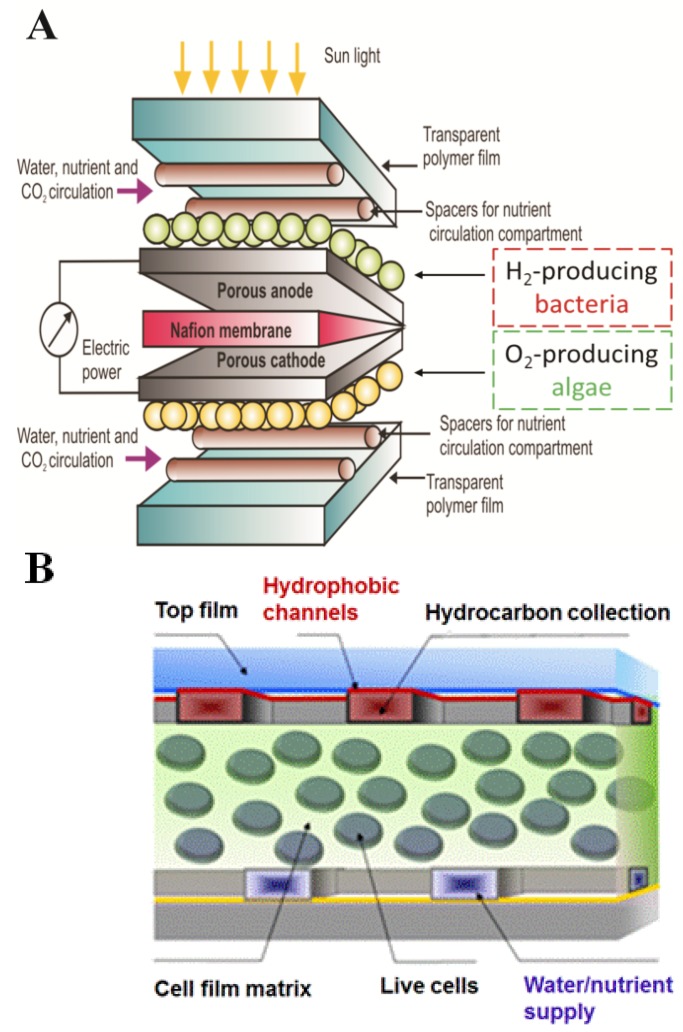
Concept of a soft, flexible photobiological fuel cell: (**A**) constructed from biocoatings of hydrogen-producing photosynthetic bacteria and oxygen-producing algae (O. Velev, V. Paunov, M. Flickinger and modified by J. Jenkins); (**B**) engineered with microfluidic channel network to provide nutrients to immobilized live cells while simultaneously separating hydrophobic product (Velev Group, NCSU, Raleigh, NC, USA).

In our contrasting colloid-based approach, we fabricated convectively-assembled thin, adhesive biocomposite algae coatings capable of being dried and rehydrated that sustained hydrogen evolution for over 100 hours [23]. Optimization of the incident light intensity [95], combined with further development of the media [57], will likely prolong the useful life of the algal coatings. These coatings prove that intact cells stabilized in thin polymer coatings have the potential to be robust components of advanced light harvesting materials for solar energy generation and carbon dioxide recycling, thus paving the way for the next generation of biomimetic leaf soft materals with multi-layer architecture engineered for light absorption and energy conversion to produce current, fuel precursors, or liquid fuels (Figure 11).

## 5. Conclusions

The major challenges facing future engineering of cellular photocomposites are the efficient use of incident solar energy, robustness, fabrication from inexpensive components and reactive half lives of years. While photosynthetic cells can be genetically engineered by synthetic and systems biology approaches for better photo efficiency [2,95], a more direct approach is to fabricate coatings containing multiple types of phtoreactive cells or multiple species, such as co-deposition of algae and photosynthetic bacteria or cyanobacteria, that absorb light in complementary wavelenths. Algae and bacteria capture light in different regions of the electromagnetic spectrum, suggesting that cellular composites could improve light utilization efficiency through multi-wavelength light harvesting. We have recently demonstrated the validity of this hypothesis by showing that a convectively-assembled, multispecies coating (one algae coating and one photosynthetic bacteria coating placed in the same tube) evolves a comparable amount of hydrogen from the same intensity of incident light as tubes containing only one algae or bacterial coating, suggesting that multi-species coatings indeed offer a promising approach. 

The continuous fabrication of thin, uniform biocomposite coatings that both maintain mechanical stability and preserve microbial viability and reactivity remains a challenging problem to both the coating industry and the biomaterials field [21,25]. The answer to the challenge is in multiple parts. First, a detailed understanding of how convective assembly process parameters, colloid-colloid interactions and the properties of the substrate (or support matrix), affect coating fabrication (structural uniformity, thickness, and particle packing) is needed. Our work demonstrates how the convective assembly technique can be extended to deposit bimodal particle size suspensions of latex microspheres and composite blends of polymer microspheres and cells on glass, plastic, and metal substrates and explains how substrate wettability, suspension composition, particle size ratio and surface charge affect coating assembly and microstructure. Another part of the answer is design of a coating apparatus for continuous fabrication of large surface area coatings without the loss of structural uniformity and the coating thinness obtained by batch CSA. We have addressed this challenge by introducing continuous convective-sedimentation assembly (CCSA), a deposition method that transforms the technique into a scalable continuous process, giving rise to larger surface area and longer coatings, by constantly supplying coating suspension to the meniscus [23,27]. To answer the question of how CCSA affects structural uniformity and thinness, the parameter investigations of batch convective assembly systems are extended to particle concentration, fluid flow-path sonication, suspension density, and meniscus volume [23]. The solution’s final component is a coating system (engineered cells, latex particles, and substrate) that permanently preserves the biosynthetic capabilities of non-growing photoreactive cells, without inhibiting viability and reactivity following composite drying and rehydration. 

## References

[1] Gosse J., Chinn M., Grunden A., Bernal O., Jenkins J., Yeager C., Kosourov S., Seibert M., Flickinger M. (2012). A versatile method for preparation of hydrated microbial-latex biocatalytic coatings for gas absorption and gas evolution. J. Ind. Microbiol. Biotechnol..

[2] Gosse J.L., Engel B.J., Hui J.C., Harwood C.S., Flickinger M.C. (2010). Progress toward a biomimetic leaf: 4000 h of hydrogen production by coating-stabilized nongrowing photosynthetic *Rhodopseudomonas palustris*. Biotechnol. Prog..

[3] Gosse J.L., Engel B.J., Rey F.E., Harwood C.S., Scriven L.E., Flickinger M.C. (2007). Hydrogen production by photoreactive nanoporous latex coatings of nongrowing *Rhodopseudomonas palustris* CGA009. Biotechnol. Prog..

[4] Ghirardi M.L., Dubini A., Yu J., Maness P. (2009). Photobiological hydrogen-producing systems. Chem. Soc. Rev..

[5] Pandit A., de Groot H., Holzwarth A. (2006). Harnessing Solar Energy for the Production of Clean Fuels.

[6] Melis A., Zhang L., Forestier M., Ghirardi M.L., Seibert M. (2000). Sustained photobiological hydrogen gas production upon reversible inactivation of oxygen evolution in the green alga *Chlamydomonas*
*reinhardtii*. Plant Physiol..

[7] Efimenko K., Finlay J., Callow M.E., Callow J.A., Genzer J. (2009). Development and testing of hierarchically wrinkled coatings for marine antifouling. ACS Appl. Mater. Interfaces.

[8] Bennett R.F., de Mora S.J. (1996). Industrial manufacture and applications of tributyltin compounds. Tributyltin: Case Study of an Environmental Contaminant.

[9] Xu Q., Barrios C., Cutright T., Newby B. (2005). Assessment of antifouling effectiveness of two natural product antifoulants by attachment study with freshwater bacteria. Environ. Sci. Pollut. Res..

[10] Weiss K.D. (1997). Paint and coatings: A mature industry in transition. Prog. Poly. Sci..

[11] Lyngberg O., Solheid C., Charaniya S., Ma Y., Thiagarajan V., Scriven L., Flickinger M. (2005). Permeability and reactivity of *Thermotoga maritima* in latex bimodal blend coatings at 80 °C: A model high temperature biocatalytic coating. Extremophiles.

[12] Swope K.L. (1995). Manipulation of Specific Enzyme Activity in Recombinant *E. coli* after Immobilization in Thin Copolymer Films: A Model System for Extended Biocatalysis. Ph.D. Thesis.

[13] Swope K., Flickinger M.C. (1996). Activation and regeneration of whole cell biocatalysts: Initial and periodic induction behavior in starved *Escherichia coli* after immobilization in thin synthetic filmss. Biotechnol. Bioeng..

[14] Piskorska M., Soule T., Gosse J.L., Milliken C., Flickinger M.C., Smith G.W., Yeager C.M. (2013). Preservation of H_2_ production activity in nanoporous latex coatings of *Rhodopseudomonas palustris* CGA009 during dry storage at ambient temperatures. Microb. Biotechnol..

[15] Julsing M.K., Kuhn D., Schmid A., Bühler B. (2011). Resting cells of recombinant *E. coli* show high epoxidation yields on energy source and high sensitivity to product inhibition. Biotechnol. Bioeng..

[16] Philips E.J., Mitsui A. (1986). Characterization and optimization of hydrogen production by a salt water blue-green alga *oscillatoria* sp. Miami BG 7. II. Use of immobilization for enhancement of hydrogen production. Int. J. Hydrog. Energy.

[17] Bagai R., Madamwar D. (1999). Long-term photo-evolution of hydrogen in a packed bed reactor containing a combination of *Phormidium valderianum*, *Halobacterium halobium*, and *Escherichia coli* immobilized in polyvinyl alcohol. Int. J. Hydrog. Energy.

[18] Dickson D.J., Page C.J., Ely R.L. (2009). Photobiological hydrogen production from *Synechocystis* sp. PCC 6803 encapsulated in silica sol-gel. Int. J. Hydrog. Energy.

[19] Leonard A., Dandoy P., Danloy E., Leroux G., Meunier C.F., Rooke J.C., Su B.-L. (2011). Whole-cell based hybrid materials for green energy production, environmental remediation and smart cell-therapy. Chem Soc. Rev..

[20] Flickinger M.C., Schottel J.L., Bond D.R., Aksan A., Scriven L.E. (2007). Painting and printing living bacteria: Engineering nanoporous biocatalytic coatings to preserve microbial viability and intensify reactivity. Biotechnol. Prog..

[21] Flickinger M.C., Fidaleo M., Gosse J., Polzin K., Charaniya S., Solheid C., Lyngberg O.K., Laudon M., Ge H., Schottel J.L., Provder T., Baghdachi J. (2009). Engineering nanoporous bioactive smart coatings containing microorganisms: fundamentals and emerging applications. Smart Coatings II.

[22] Jerrim L.B., Velev O.D. (2009). Deposition of coatings from live yeast cells and large particles by “convective-sedimentation” assembly. Langmuir.

[23] Jenkins J.S., Flickinger M.C., Velev O.D. (2013). Continuous convective-sedimentation assembly of colloidal microsphere coatings for biotechnology applications. Coatings.

[24] Fidaleo M., Flickinger M.C. (2011). Engineering and modeling of thin, adhesive, microbial biocatalytic coatings for high intensity oxidations in multi-phase microchannel bioreactors. Chem. Eng. Sci..

[25] Scriven L.E.S., Galan M.A., del Valle M. (2005). Fine–structured materials by continuous coating and drying or curing of liquid precursors. Chemical Engineering.

[26] Lyngberg O., Ng C., Thiagarajan V., Scriven L., Flickinger M. (2001). Engineering the microstructure and permeability of thin multilayer latex biocatalytic coatings containing *E.*
*Coli*. Biotechnol. Prog..

[27] Jenkins J.S., Flickinger M.C., Velev O.D. (2012). Deposition of composite coatings from particle-particle and particle-yeast blends by convective-sedimentation assembly. J. Colloid Interface Sci..

[28] Kuncicky D.M., Naik R.R., Velev O.D. (2006). Rapid deposition and long-range alignment of nanocoatings and arrays of electrically conductive wires from tobacco mosaic virus. Small.

[29] Kumnorkaew P., Ee Y., Tansu N., Gilchrist J.F. (2008). Investigation of the deposition of microsphere monolayers for fabrication of microlens arrays. Langmuir.

[30] Prevo B., Velev O. (2004). Controlled rapid deposition of structured coatings from micro-and nanoparticle suspensions. Langmuir.

[31] Hosein I.D., Lee S.H., Liddell C.M. (2010). Dimer-based three-dimensional photonic crystals. Adv. Funct. Mater..

[32] Hosein I.D., Liddell C.M. (2007). Convectively assembled nonspherical mushroom cap-based colloidal crystals. Langmuir.

[33] Chen K., Stoianov S.V., Bangerter J., Robinson H.D. (2010). Restricted meniscus convective self-assembly. J. Colloid Interface. Sci..

[34] Kim M., Im S., Park O. (2005). Rapid fabrication of two- and three-dimensional colloidal crystal films via confined convective assembly. Adv. Funct. Mater..

[35] Dimitrov A., Nagayama K. (1996). Continuous convective assembling of fine particles into two-dimensional arrays on solid surfaces. Langmuir.

[36] Denkov N., Velev O., Kralchevsky P., Ivanov I., Yoshimura H., Nagayama K. (1992). Mechanism of formation of two-dimensional crystals from latex particles on substrates. Langmuir.

[37] Denkov N., Velev O., Kralchevsky P., Ivanov I., Yoshimura H., Nagayama K. (1993). Two-dimensional crystallization. Nature.

[38] Dimitrov A, Nagayama K. (1995). Steady-state unidirectional convective assembling of fine particles into two-dimensional arrays. Chem. Phys. Lett..

[39] Jenkins J.S. (2013). Engineering Multifunctional Living Paints: Thin, Convectively-Assembled Biocomposite Coatings of Live Cells and Colloidal Latex Particles Deposited by Continuous Convective-Sedimentation Assembly. Ph.D. Thesis.

[40] Prevo B., Hwang Y., Velev O. (2005). Convective assembly of antireflective silica coatings with controlled thickness and refractive index. Chem. Mater..

[41] Prevo B., Fuller J., Velev O. (2005). Rapid deposition of gold nanoparticle films with controlled thickness and structure by convective assembly. Chem. Mater..

[42] Kuncicky D., Christesen S., Velev O. (2005). Role of the micro- and nanostructure in the performance of SERS substrates assembled from gold nanoparticles. Appl. Spectrosc..

[43] Lawton C.W., Klei H.E., Sundstrom D.W., Voronoko P.J. (1986). Immobilization of whole cells using polymeric coatings. Biotechnol. Bioeng. Symp..

[44] Lawton C.W. (2001). *Saccharomyces Cerevisiae* Immobilization Using Latex Polymers. M.S. Thesis.

[45] Bunning T.J. (1988). Physical Property Improvements of A Biocatalyst. M.S. Thesis.

[46] Schaffer J.R., Burdick B.A., Abrams C.T. (1988). Thin-film biocatalysts. Chemtech.

[47] Flanagan W.P., Klei H.E., Sundstrom D.W., Lawton C.W. (1990). Optimization of a pellicular biocatalyst for penicillin G production by *Penicillium chrysogenum*. Biotechnol. Bioeng..

[48] Cantwell J.B., Mills P.D.A., Jones E., Stewart R.F. (1988). Immobilized cells. Eur. Pat..

[49] Martens N., Hall E.A.H. (1994). Immobilisation of photosynthetic cells based on film-forming emulsion polymers. Anal. Chim. Acta.

[50] Pansu B., Pieranski P., Pieranski P. (1984). Structures of thin layers of hard spheres: High pressure limit. J. Phys..

[51] Pansu B., Pieranski P., Strzelecki L. (1983). Thin colloidal crystals: A series of structural transitions. J. Phys..

[52] Tessier P., Velev O., Kalambur A., Rabolt J., Lenhoff A., Kaler E. (2000). Assembly of gold nanostructured films templated by colloidal crystals and use in surface-enhanced Raman spectroscopy. J. Am. Chem. Soc..

[53] Gupta S., Alargova R.G., Kilpatrick P.K., Velev O.D. (2010). On-chip dielectrophoretic co-assembly of live cells and particles into responsive biomaterials. Langmuir.

[54] Prevo B.G., Kuncicky D.M., Velev O.D. (2007). Engineered deposition of coatings from nano- and micro-particles: A brief review of convective assembly at high volume fraction. Colloid Surf. A Physicochem. Eng. Asp..

[55] Fakhrullin R.F., Brandy M., Cayre O.J., Velev O.D., Paunov V.N. (2010). Live celloidosome structures based on the assembly of individual cells by colloid interactions. Phys. Chem. Chem. Phys..

[56] Brandy M., Cayre O.J., Fakhrullin R.F., Velev O.D., Paunov V.N. (2010). Directed assembly of yeast cells into living yeastosomes by microbubble templating. Soft Matter.

[57] Kosourov S.N., Seibert M. (2009). Hydrogen photoproduction by nutrient-deprived *Chlamydomonas reinhardtii* cells immobilized within thin alginate films under aerobic and anaerobic conditions. Biotechnol. Bioeng..

[58] Tredici M.R., Flickinger M.C., Drew S.W. (1999). Bioreactors. The Encyclopedia of Bioprocess Technology: Fermentation, Biocatalysis, and Bioseparation.

[59] Melo L.F., Bott T.R. (1992). Biofilms: Science and Technology.

[60] Bryers J.D. (2000). Biofilms II: Process Analysis and Applications.

[61] Characklis W.G., Marshall K.C. (1990). Biofilms.

[62] Evans L.V. (2000). Biofilms: Recent Advances in Their Study and Control.

[63] Donlan R. (2002). Biofilms: Microbial life on surfaces. Emerg. Infect. Dis..

[64] Burgess J., Boyd K., Armstrong E., Jiang Z., Yan L., Berggren M., May U., Pisacane T., Granmo Å., Adams D. (2003). The development of a marine natural product-based antifouling paint. Biofouling.

[65] Blossey R. (2003). Self-cleaning surfaces—Virtual realities. Nat. Mater..

[66] Haines R.S., Wu A.H.F., Zhang H., Coffey J., Huddle T., Lafountaine J.S., Lim Z.-J., White E.A., Tuong N.T., Lamb R.N. (2009). Self-cleaning surfaces: A third-year undergraduate research project. J. Chem. Educ..

[67] Stamm L.B.J. (2009). Meniscus-Directed Assembly of Biologically Active Coatings of Cells, Microparticles, and Nanoparticles. Ph.D. Thesis.

[68] Zhou H., Fan T., Zhang D. (2011). An insight into artificial leaves for sustainable energy inspired by natural photosynthesis. ChemCatChem.

[69] Bensaid S., Centi G., Garrone E., Perathoner S., Saracco G. (2012). Towards artificial leaves for solar hydrogen and fuels from carbon dioxide. ChemSusChem.

[70] Nocera D.G. (2010). The artificial leaf. Acc. Chem. Res..

[71] Blankenship R.E., Tiede D.M., Barber J., Brudvig G.W., Fleming G., Ghirardi M., Gunner M.R., Junge W., Kramer D.M., Melis A. (2011). Comparing photosynthetic and photovoltaic efficiencies and recognizing the potential for improvement. Science.

[72] Lee M., Kim J.H., Lee S.H., Park C.B. (2011). Biomimetic artificial photosynthesis by light-harvesting synthetic wood. ChemSusChem..

[73] Rooke J.C., Meunier C., Leonard A., Su B. (2008). Energy from photobioreactors: Bioencapsulation of photosynthetically active molecules, organelles, and whole cells within biologically inert matrices. Pure Appl. Chem..

[74] Jain S.L., Joseph J.K., Kuehn F.E., Reiser O. (2009). Retraction: An efficient synthesis of poly(ethylene glycol)-supported iron(II) porphyrin using a click reaction and its application for the catalytic olefination of aldehydes. Adv. Synth. Catal..

[75] Frolov L., Wilner O., Carmeli C., Carmeli I. (2008). Fabrication of oriented multilayers of photosystem I proteins on solid surfaces by auto-metallization. Adv. Mater..

[76] Krassen H., Schwarze A., Friedrich B., Ataka K., Lenz O., Heberle J. (2009). Photosynthetic hydrogen production by a hybrid complex of photosystem I and [NiFe]-hydrogenase. ACS Nano..

[77] Branyik T., Kuncova G., Paca J., Demnerova K. (1998). Encapsulation of microbial cells into silica gel. J. Sol. Gel. Sci. Technol..

[78] Finnie K., Bartlett J., Woolfrey J. (2000). Encapsulation of sulfate-reducing bacteria in a silica host. J. Mat. Chem..

[79] Livage J., Coradin T., Roux C. (2001). Encapsulation of biomolecules in silica gels. J. Phys. Condens. Matter..

[80] Fennouh S., Guyon S., Livage J., Roux C. (2000). Sol-gel entrapment of *Escherichia coli*. J. Sol. Gel. Sci. Technol..

[81] Ferrer M., Yuste L., Rojo F., del Monte F. (2003). Biocompatible sol-gel route for encapsulation of living bacteria in organically modified silica matrixes. Chem. Mater..

[82] Bottcher H., Soltmann U., Mertig M., Pompe W. (2004). Biocers: Ceramics with incorporated microorganisms for biocatalytic, biosorptive and functional materials development. J. Mat. Chem..

[83] Fiedler D., Thron A., Soltmann U., Bottcher H. (2004). New packing materials for bioreactors based on coated and fiber-reinforced biocers. Chem. Mater..

[84] Rooke J.C., Leonard A., Sarmento H., Meunier C.F., Descy J., Su B. (2011). Novel photosynthetic CO_2_ bioconvertor based on green algae entrapped in low-sodium silica gels. J. Mat. Chem..

[85] Meunier C.F., Rooke J.C., Leonard A., van Cutsem P., Su B. (2010). Design of photochemical materials for carbohydrate production via the immobilisation of whole plant cells into a porous silica matrix. J. Mater. Chem..

[86] Leino H., Kosourov S.N., Saari L., Sivonen K., Tsygankov A.A., Aro E., Allahverdiyeva Y. (2012). Extended H_2_ photoproduction by N_2_-fixing cyanobacteria immobilized in thin alginate films. Int. J. Hydrog. Energy.

[87] Buchholz K., Kasche V., Bornscheuer U.T. (2005). Biocatalysis and Enzyme Technology.

[88] Webb C., Dervakos G.A. (1996). Studies in Viable Cell Immobilization.

[89] Karel S., Robertson C. (1989). Autoradiographic determination of mass-transfer limitations in immobilized cell reactors. Biotechnol. Bioeng..

[90] Karel S., Robertson C. (1989). Cell mass synthesis and degradation by immobilized *Escherichia coli*. Biotechnol. Bioeng..

[91] De Bont J.A.M., Visser J., Mattiassen B., Tramper J. (1990). Physiology of Immobilized Cells.

[92] Willert R.G., Baron G.V., de Backer L. (1996). Immobilized Living Cells: Modeling and Experimental Methods.

[93] Guisan J.M. (2006). Immobilization of Enzymes and Cells.

[94] Wijffels E.H. (2001). Immobilized Cells.

[95] Kosourov S.N., Ghirardi M.L., Seibert M. (2011). A truncated antenna mutant of *Chlamydomonas reinhardtii* can produce more hydrogen than the parental strain. Int. J. Hydrog. Energy.

